# Dr. Darwall

**Published:** 1833-10-01

**Authors:** 


### Obituary.

### Dr. Darwall.

We have the melancholy task of recording the death, on Saturday, the 10th of August, after a short illness, of this eminent and respected physician; which lamented occurrence was occasioned by an injury received in the examination of a dead body, at the Birmingham Hospital, on Tuesday, the 30th July. During that day, Dr. Darwall was unsuspicious of having incurred any danger. He pursued his usual avocations —visited his patients—and, in the evening, walked in the Botanic Garden. Very early in the morning of Wednesday he was attacked with severe shivering, to which violent re-action soon succeeded, with inflammation of the absorbents of his left hand and arm. Although these symptoms subsided, under the skilful attentions of the professional friends who were soon assembled round him, his system never recovered the shock; and after eleven anxious days, during most of which sanguine hopes were entertained of his recovery, he breathed his last, in the 40th year of his age. The shortness of his last illness—its accidental origin —and the abrupt cessation of his extraordinary mental activity, are circumstances so painfully afflicting to his family and to his immediate friends, that even the very general concern which has been occasioned by these sad events, cannot, for the present, be expected to soften the poignancy of their sorrow. If great mental acuteness—if extensive learning—if a profound knowledge of medicine, united with the highest practical skill—if industry the most indefatigable, and benevolence no less enlightened than it was ardent, could have been any protection from an early death, the public might yet, for many years, have benefited by his ability and his experience.

For about two years before his death, he held the office of physician to the General Hospital—long an object of his ambition, and to which he was elected by the Governors with the most flattering marks of consideration; individuals of all parties concurring in the appoinment, and more than one of his medical competitors seceding from all opposition to claims which rested on every merit that could entitle a medical practitioner to distinction. One of his first steps after his appointment, was to commence a series of lectures on such cases as he deemed the most instructive; from which, if his life had been prolonged, the pupils of the Hospital would doubtless have derived great advantage. His work on the Diseases of Children, published about three years since, is characterized by all that should distinguish the production of a practical physician; and the remarks on Spinal Irritation, appended to it, excited very general attention from the profession. He had proceeded some way in a History of Medicine, the first part of which is, we believe, about to be published under the superintendence of the "Society for the Promotion of Useful Knowledge." His contributions to the *Cyclopædia of Practical Medicine*, on the Diseases of Artizans, and on the different varieties of Dropsy, possess the highest value. He was engaged in the preparation of some papers on the Curability of Consumption, intended for the Transactions of the "Provincial Medical and Surgical Association;" which, as they would have been, like all his writings, the result of long observation and a sound judgment, it is to be hoped may be found sufficiently advanced for publication. At the late great meeting of medical men, held at Bristol, he was unanimously appointed to deliver the Oration at the next anniversary of the Association, to be held at Birmingham, in July, 1834.

His opinions on some of the most important questions which divide the sentiments of mankind might be considered as carried to some extreme; and easier natures might reproach him with being too much a prey to care and anxiety. These (if faults they were) were the only faults which clung to him. But those who knew him best well knew, that even in these respects, what seemed blameable to such as knew him less, did but arise out of the uprightness of his mind, and his scrupulous conscientiousness. In him, what are called High Tory and High Church principles, had not effaced any of the best feelings of humanity, or interfered with his friendships. He *spoke* openly, and boldly, and at all times; but to *do* any thing cruel and unkind was not in his disposition. He felt warmly for what he conceived to be the best interests of his country; and he despised all the various disguises of political dishonesty. His piety also was unaffected, but fervent and sincere, and influenced all his thoughts and actions. His opinions on all points of policy connected with his own profession were in the highest degree liberal and judicious. And if, as regarded the management of his own mind, he too severely tasked his own powers—a severity which, perhaps, prepared him too surely to sink under accidental injury—he was ever considerate and indulgent towards those to whom such incessant exertions were impossible.

For his afflicted widow, for his bereaved children, for his grieving friends, who must long mourn the loss of such a man there is some consolation in the reflection, that he whom they admired and loved, and whom they now lament, lived and died with so much honour, and a reputation so unblemished—that the longest life could have done no more than afford a longer opportunity for the exercise of virtues of which he had proved himself to be eminently possessed. But there is a further and deeper consolation in the certain conviction, which none can fail to entertain who habitually refer worldly events to their Great First Cause, that the death of this distinguished physician, like his well-spent life, was ordered by the same over-ruling Providence which guides not only the workings of the material world, but all the efforts of human minds;—wills their development—prescribes their course and direction—watches over their best exertions—permits their highest aspirations—and, when their allotted task in the great moral scheme is done, suffers them to be for a time obscured and dishonoured by weakness, and decay, and death, to rise in honour, and incorruption, and power, in another and a brighter sphere.*

* Extracted from a Memoir written, we believe, by Dr. Connolly of Warwick, and published in the Warwick Advertiser, and other provincial journals.—*Ed. Med. C. Rev.*

